# Downregulation and Hypermethylation of GABPB1 Is Associated with Aggressive Thyroid Cancer Features

**DOI:** 10.3390/cancers14061385

**Published:** 2022-03-08

**Authors:** Xiangling Xing, Ninni Mu, Xiaotian Yuan, Na Wang, C. Christofer Juhlin, Klas Strååt, Catharina Larsson, Shi Yong Neo, Dawei Xu

**Affiliations:** 1 Department of Medicine, Division of Hematology, Bioclinicum J6:20 and Center for Molecular Medicine, Karolinska University Hospital Solna and Karolinska Institutet, SE-171 64 Solna, Sweden; xiaotian.yan@ki.se (X.Y.); klas.straat@gmail.com (K.S.); dawei.xu@ki.se (D.X.); 2 Department of Oncology-Pathology, BioClinicum J6:20, Karolinska Institutet, SE-171 64 Solna, Sweden; ninni.mu@ki.se (N.M.); christofer.juhlin@ki.se (C.C.J.); shiyong.neo@ki.se (S.Y.N.); 3 Laboratory Animal Center, Shandong Provincial Hospital Affiliated to Shandong First Medical University, Jinan 250021, China; 4 Department of Medicine-Huddinge, Karolinska Institutet, SE-171 77 Stockholm, Sweden; na.wang@ki.se; 5 Department of Pathology and Cancer Diagnostics, Karolinska University Hospital-Solna, SE-171 76 Stockholm, Sweden; 6 Singapore Immunology Network, Agency for Science, Technology and Research, 8A Biomedical Grove, Singapore 138648, Singapore

**Keywords:** GABPA, GABPB1, DNA methylation, telomerase, TERT, thyroid carcinoma

## Abstract

**Simple Summary:**

Promoter mutations of the telomerase reverse transcriptase (*TERT*) gene have been suggested as an oncogenic event in various cancers, including thyroid cancer (TC). GABPB1 is reported to activate *TERT* gene expression and has been proposed as a cancer therapeutic target. The aim of this study is to explore the fate of TC cells after disruption of GABPB1 and its role in TC. We found that besides the reported oncogenic role of GABPB1 in activating *TERT*, it also has tumor-suppressive functions in TC. Therefore, targeting GABPB1 for cancer therapy should be cautious since it may counteract its tumor-suppressive functions.

**Abstract:**

Promoter mutations of the telomerase reverse transcriptase (*TERT*) gene occur frequently in thyroid carcinoma (TC), including papillary (PTC) and anaplastic subtypes (ATC). Given that the ETS family transcription factors GABPA and GABPB1 activate the mutant *TERT* promoter and induce *TERT* expression for telomerase activation, GABPB1 has been proposed as a cancer therapeutic target to inhibit telomerase. Here, we sought to determine the role of GABPB1 in TC pathogenesis. In TC-derived cells carrying the mutated *TERT* promoter, GABPB1 knockdown led to diminished *TERT* expression but significantly increased invasive potentials in vitro and metastatic potential in a xenograft zebrafish model and altered expression of markers for epithelial-to-mesenchymal transition. *GABPB1* expression was downregulated in aggressive TCs. Low *GABPB1* expression correlated with its promoter hypermethylation, which in turn was also associated with shorter disease-free survival. Consistently, DNA methylation inhibitors enhanced *GABPB1* expression, as observed upon reduced promoter methylation. Our results suggest that GABPB1 is required for *TERT* expression and telomerase activation, but itself serves as a tumor suppressor to inhibit TC progression. Furthermore, aberrant DNA methylation leads to GABPB1 silencing, thereby promoting TC aggressiveness. Thus, caution is needed if targeting GABPB1 for cancer therapy is considered.

## 1. Introduction

Telomerase is a ribonucleoprotein lengthening telomeric DNA sequences at the termini of human linear chromosomes, and telomerase reverse transcriptase (*TERT*) is a rate-limiting component of the telomerase enzyme [1,2,3,4]. In most differentiated cells, telomerase activity is absent due to the default repression of the *TERT* gene; however, *TERT* induction and telomerase activation occur widely in tumor development. The key function of TERT and telomerase is to stabilize telomere length through which transformed cells acquire an immortal phenotype [2,5]. In addition, accumulating evidence supports that TERT/telomerase exhibits oncogenic activities far beyond its telomere-lengthening action, thereby contributing to multiple hallmarks of cancer [2,3,6]. Targeting of TERT/telomerase is currently attempted for cancer therapy [7].

Multiple mechanisms have been identified to activate *TERT* transcription and telomerase in oncogenesis, among which the *TERT* promoter mutation is the most studied [2]. The mutations occur at two hotspots (−124 and −146 upstream of ATG), and both are C > T transitions, denoted C228T and C250T, respectively [8,9]. These mutations create de novo ETS binding motifs that are mainly recognized and bound by the ETS family transcription factors GABP sub-group members [10,11]. The GABP factors, including the DNA binding domain-containing GABPα (GABPA) and the trans-activation domain-bearing GABPβ (GABPB), can act as a heterodimer or heterotetramer to activate the transcription of their target genes [11,12]. While GABPA has a single isoform encoded by the *GABPA* gene, GABPB has several isoforms encoded by two distinct genes, either *GABPB1* or *GABPB2* [10,13]. *GABPB1* encodes GABPB1 with four isoforms, including two long GABPB1L and two short GABPB1S members. *GABPB2* encodes GABPB2, which has a single isoform [11,12]. The GABPB1 and GABPB2 isoforms are highly homologous, sharing a common N-terminus containing the ankyrin repeat (AR) domain that mediates the interaction with GABPA. Whereas GABPB1S is only functional to dimerize with GABPA, both GABPB1L and GABPB2 contain highly homologous C-terminal leucine-zipper domains that mediate formation of homodimerization or heterodimerization [12,14]. However, the GABPA-GABPB1 complex was shown to be more relevant with regulation of mutant *TERT* promoter or *TERT* expression [13,15]. More importantly, GABPB1 knockout inhibits *TERT* expression and telomerase activity in *TERT* promoter-mutated glioblastoma cells, disrupting telomere length maintenance through which apoptosis is induced [13]. Therefore, telomerase/TERT repression by inhibiting GABPB1 expression was proposed as a therapeutic strategy against cancer carrying a mutated *TERT* promoter [13].

*TERT* promoter mutations occur preferably in certain malignancies, including thyroid carcinomas (TCs) [16]. TCs are in general classified into papillary (PTC), follicular (FTC), medullary (MTC), poorly differentiated (PDTC), and anaplastic (ATC) subtypes, with PTC as the predominant subtype (>80%). We and others have previously observed that approximately 10–25% of PTCs carry *TERT* promoter mutations and that their presence predicts poor patient outcomes [16,17,18,19,20,21]. In addition, the activating *BRAF*V600E mutation occurs frequently in PTC tumors, and the coexistence of *TERT* promoter mutations with *BRAF*V600E has been shown to be over-represented in the most aggressive PTCs and also in cases with dedifferentiation to ATCs [17,18,22,23]. Mechanistically, Liu et al. demonstrated that the hyperactive BRAF-MAP kinase cascade leads to the phosphorylation and activation of the transcription factor FOS, which in turn increases the expression of GABPB1, thereby driving formation of the GABPA-GABPB1 complex to activate mutant *TERT* promoter for *TERT* expression in a panel of human cancers including TC [24]. Moreover, FOS inhibitors were recently observed to induce robust apoptosis in cancer cells harboring a mutant *TERT* promoter by suppressing GABPB1 and *TERT* expression [25].

The observations above indicate that GABPB1 may act as a key mediator linking two PTC-related oncogenic signals. However, the fate of TCs upon GABPB1 dysregulation, as well as its role in TC pathogenesis, is still unclear. Unexpectedly, the findings presented herein reveal that GABPB1 knockdown leads to more aggressive behaviors of TC cells, despite impaired *TERT* expression. Consistently, the downregulation of *GABPB1* expression was associated with aggressive TC tumors, and low *GABPB1* mRNA correlated with *GABPB1* promoter hypermethylation, which was associated with shorter disease-free survival. To our knowledge, the present study is the first to identify the epigenetic control of *GABPB1*, highlighting how cancer-associated DNA methylation downregulates *GABPB1* to decrease its tumor-suppressing functions during TC development.

## 2. Materials and Methods

### 2.1. Cell Lines and Cell Culturing

The study included three TC-derived cell lines U-hth-74, U-hth-104, and MDA-T41. Cells were cultured in RPMI-1640 medium (Thermo Fisher Scientific, Waltham, MA, USA) supplemented with 10% fetal bovine serum (FBS) (Thermo Fisher Scientific), 100 U/mL penicillin, 100 μg/mL streptomycin, and 4 mM L-glutamine. The ATC-derived U-hth-74 and U-hth-104 cell lines carry a C228T TERT promoter mutation as verified by Sanger sequencing [19], while the PTC-derived MDA-T41 carries a wild-type (WT) TERT promoter [26]. For DNA methylation inhibition, U-hth-74 and U-hth-104 cells seeded in 6-well plates were treated with 10 μM of 5-Azacytidine (5-Aza, Sigma-Aldrich, Darmstadt, Germany) for 72 h. During the 72 h of culturing, medium was changed every 24 h. Control cells were incubated with the same volume of DMSO. ATC-derived cell lines U-hth-74 and U-hth-104 were obtained from Dr. N-E Heldin and short tandem repeats (STR) genotyping was previously performed and matched to previously published genotypes [19,27]. MDA-T41 was purchased from the American Type Culture Collection (ATCC, Manassas, VA, USA). All cell experiments were repeated three times unless stated otherwise.

### 2.2. Patients and Tumor Specimens

Fresh frozen primary tumor specimens were obtained from the Karolinska University Hospital Biobank, including 93 cases of papillary thyroid carcinomas (PTC_K_) operated between 1987 and 2005 and 18 cases of anaplastic thyroid carcinomas (ATC_K_) operated between 1989 and 2007. Patient inclusion and exclusion criteria and sample preparation have been previously described in detail [27]. In short, tumor samples were collected after surgery according to an established procedure and kept frozen at −70 °C until use. Patients were classified according to the 2004 World Health Organization (WHO) classification of endocrine tumors, and cases diagnosed as a follicular variant of PTC (FV-PTC) were excluded from the study to avoid inclusion of non-invasive follicular thyroid neoplasms with papillary-like nuclear features (NIFTP) according to the 2017 WHO classification [28]. Cases were reviewed by an experienced pathologist (C.C.J.) to determine tumor cell representation. Clinical information was retrospectively collected for the PTC_K_ cohort, including age at diagnosis, sex, tumor size, lymph node metastases, and distant metastasis. In addition, follow-up data on overall survival (endpoints: dead or alive) and disease-free survival (endpoints: relapsed/progression or disease-free) were registered for PTC_K_ patients. Ethical permission was obtained from the Swedish Ethical Review Authority, and informed consent was given prior to sample collection.

### 2.3. The Cancer Genome Atlas (TCGA) Cohort of PTC

The clinico-pathological information, genetic, RNA expression, and methylation data for the TCGA cohort of 393 PTC (PTC_TCGA_) were downloaded via cBioPortal (http://www.cbioportal.org, accessed on 5 January 2021) or GDC Data Portal (https://portal.gdc.cancer.gov, accessed on 5 January 2021). In addition, expression and methylation data were downloaded for non-tumorous adjacent thyroid tissue samples from 56 cases used as normal thyroid controls (NC_TCGA_). mRNA abundances were expressed as RSEM (RNA-Seq by expectation maximization). DNA methylation was expressed as β values (the ratio of signal intensity between methylated and unmethylated CpGs). The thresholds of 0.2 and 0.8 (β values) were applied to define hypomethylation and hypermethylation, respectively, with the in-between values indicating intermediate levels of methylation [29,30]. For TCGA pan-cancer analysis, batch-effects normalized gene expression (log2 expression) and methylation 450 K (β value) of various CpG sites within the *GABPB1* gene locus were downloaded via UCSC Xena browser (xena.ucsc.edu). Similarly, FV-PTC tumor subtypes were excluded from the thyroid cancer cohort (THCA). Methylation densities across 33 cancer types were presented in an unclustered heatmap using the “pheatmap” R package (version 1.0.12). Tukey boxplots were created with customized codes from the “ggplot” R package (version 3.3.5).

### 2.4. Sanger Sequencing

The *BRAF*V600E and hotspot *TERT* promoter (C228T and C250T) mutation status were analyzed by Sanger sequencing with primers listed in Table S1 in our patient cohorts and cell lines as presented in our previous study [19].

### 2.5. RNA Interference (RNAi) Transfection

GABPB1 or GABPA RNAis (IDT) were transfected into cells with Lipofectamine2000 (Thermo Fisher Scientific) according to the protocol provided by the manufacturer. Sequences for these RNAis are listed in Table S1.

### 2.6. Gene Expression by Quantitative Real-Time PCR (qPCR)

Gene expression levels of *TERT*, *DICER1*, *GABPA,* and *GABPB1* were quantified in cell lines and tumor samples by qPCR with Taqman Gene expression assays or SYBR Green (Table S1) using QuantStudio 7 Flex Real-Time PCR System (Thermo Fisher Scientific). Gene expressions were normalized to a house-keeping gene (*B2M* or *ACTB*), and relative expressions were calculated based on ΔCT values.

### 2.7. Cellular Invasion Assays

A total of 50 μL of matrigel (Corning Life Sciences, Flintshire, U.K.) was first loaded to the bottom of the upper chamber, and cells (2.5 × 10^4^) were then seeded into the upper chamber. The lower chamber contained RPMI-1640 medium with 20% FBS. Cells passing through the matrigel were stained with crystal violet, photographed, and counted 48 h later.

### 2.8. Cell Proliferation Analyses

Proliferation of the GABPB1-depleted U-hth-74 and U-hth-104 cells was monitored and analyzed every 8 h for a total of 72 h using an IncuCyte S3 Live-Cell Analysis System (Essen Bioscience, Ann Arbor, MI, USA). The changes of phase area confluence represent cell proliferation.

### 2.9. Flow Cytometry

For apoptosis assays, FITC Annexin V (Biolegend, San Diego, CA, USA, 640905) was used to stain cells, and fluorescence signals were determined. To detect markers of epithelial-to-mesenchymal transition (EMT), anti-Vimentin antibody (BD Biosciences, San Diego, CA, USA, 562338) and anti-E-Cadherin antibody (Biolegend, 324114) were used. LIVE/DEAD™ Fixable Aqua Dead Cell Stain Kit (Thermo Fisher Scientific, L34965) was used to stain viable cells in both apoptosis and EMT phenotyping. All data were acquired on Novocyte Quanteon 4020 (Agilent Technologies, Santa Clara, CA, USA) and analyzed with FlowJo v10 software (BD Biosciences).

### 2.10. Zebrafish Husbandry and Injection Experiments

Zebrafish were housed in self-cleaning 3.5 L tanks with a density of 5 fish per liter in a centralized recirculatory aquatic system (Tecniplast, West Chester, PA, USA). Basic water parameters were continuously surveilled and automatically adjusted to a temperature of 28 °C; conductivity 1200 µS/cm, pH 7.5. Other chemical water parameters were checked minimum monthly. The lightning scheme was 14 h light/10 h dark with a 20 min dusk and dawn period. Health monitoring using sentinel fish was performed quarterly (IDEXX Bioanalytics). Zebrafish embryos were raised in E3 medium in a light-cycle incubator and staged according to Kimmel et al. [31]. All husbandry procedures followed standardized operation procedures, which are available on request.

Zebrafish AB embryos were anesthetized with 160 µg/mL tricaine (Sigma, adjusted to pH 7.2 with Na2HPO4) and placed on a flat agarose surface. U-hth-104 cells were stained with CellTracker™ CM-DiI Dye (Thermo Fisher Scientific, C7000) and suspended before loading into microcapillaries without filament (World Precision Instruments, Sarasota, FL, USA), and about 150 cancer cells were injected into the perivitelline space of 48 h postfertilization (hpf) embryos using a Femtojet 4X micropump. Successfully injected embryos were selected and placed into a 96-well imaging plate (IBIDI, 89621) in which an agarose bed had been casted using a 3D-printed orientation tool [32]. The plate was imaged using an ImageXpress Nano (Molecular Devices, Silicon Valley, CA, USA).

### 2.11. Pyrosequencing for DNA Methylation Analyses

Genomic DNA was extracted using QIAamp DNA Blood Mini Kit (Qiagen, Hilden, Germany) and then converted by Sodium Bisulfite using EpiTect Bisulfite Kit (Qiagen). PCR amplification was performed with *GABPB1* promoter-specific primers. The PCR product was purified by binding to streptavidin-coated sepharose beads (GE Healthcare, Chicago, IL, USA), denatured, and washed. The sequencing primer was then annealed to the purified PCR fragment followed by pyrosequencing in a PyroMark Q96 (Qiagen). The primer sequences used are listed in Table S1.

### 2.12. Western Blot Analysis

Proteins were extracted using Pierce RIPA Buffer (Thermo Scientific) with 1% phenylmethanesulfonyl fluoride (Sigma-Aldrich) and quantified with BCA Protein Assay (Bio-Rad, Hercules, CA, USA). A total of 30 µg of proteins were separated in Mini-PROTEAN TGX Gels (Bio-Rad) and transferred to PVDF membranes using Trans-Blot Turbo Transfer Pack (Bio-Rad). Membranes were blocked with 5% non-fat milk diluted in TBST and then incubated with primary antibodies and secondary antibodies before being imaged with Clarity Max Western ECL Substrate (Bio-Rad, 1705062) and ChemiDoc MP Imaging System (Bio-Rad). Anti-GABPB1 (1:1000 dilution, Santa Cruz, sc-271571) and anti-β-Actin (1:50,000 dilution, Santa Cruz, sc-47778) were used as primary antibodies. Goat Anti-Mouse IgG (H+L)-HRP Conjugate (Bio-Rad, 170-6516) served as secondary antibody.

### 2.13. Statistical Analyses

All statistical analyses were performed using IBM SPSS Statistics version 24 (IBM, Armonk, NY, USA) or GraphPad Prism 8 (GraphPad Software, San Diego, CA, USA). Based on the distribution of data, Student’s *t*-test, Mann–Whitney *U*-test, Kruskal–Wallis test, and chi2-test or Fisher’s exact test were used for comparison between groups. Spearman’s rank-order correlation coefficient was applied to determine correlation coefficient r. Survival analyses were performed with log-rank test. Overall and disease-free survivals were visualized with Kaplan–Meier plots. *p*-values < 0.05 were considered as statistically significant.

## 3. Results

### 3.1. Reduced TERT Expression Coupled with Enhanced Invasion in GABPB1-Depleted TC Cells

**Figure 1:**
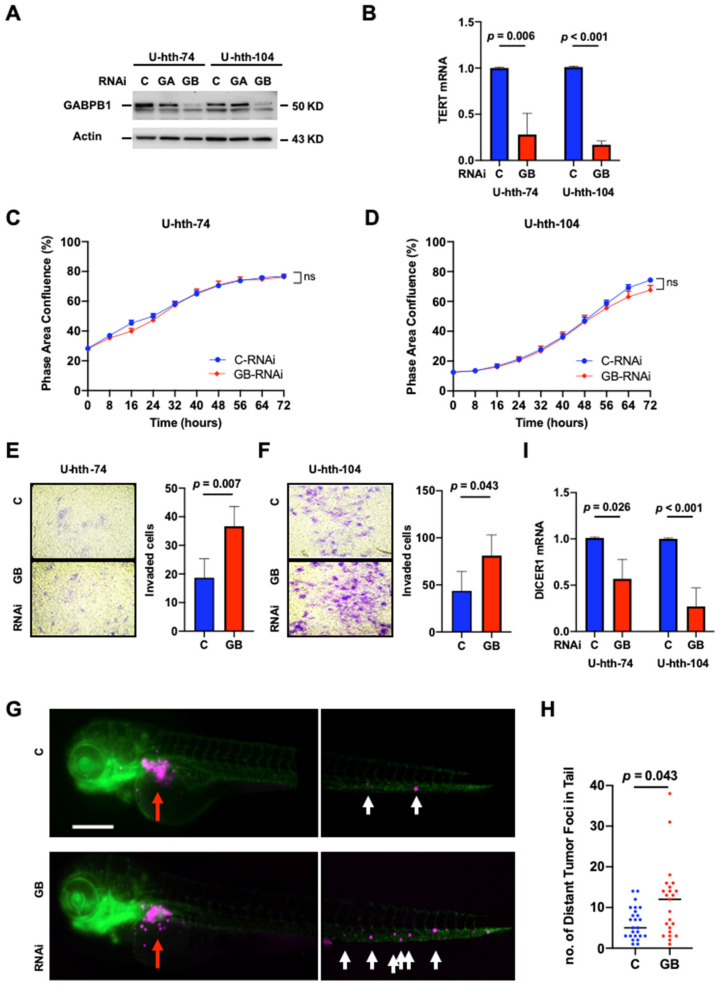
GABPB1 depletion leads to diminished *TERT* and *DICER1* expression but promotes invasiveness and distant metastasis of TC cells. U-hth-74 and U-hth-104 cells were transfected with control and GABPB1 RNAis, respectively, and harvested for analyses at 48 h. Three independent experiments were performed. (**A**) Verification of GABPB1 knockdown efficiency using immunoblotting. RNAi to GABPA was also included as an additional control to support the RNAi specificity. Original Images for Western blots are shown in Figure S7. (**B**) Downregulation of *TERT* mRNA expression in GABPB1-depleted cells. qPCR was performed to determine mRNA levels. (**C**,**D**) Proliferation analyses of GABPB1-depleted U-hth-74 and U-hth-104 cells. (**E**,**F**) Enhanced invasion of GABPB1-depleted U-hth-74 and U-hth-104 cells. Matrigel assays were used to determine invasive potentials of cells. (**G**) Knocking down of GABPB1 in U-hth-104 cells promotes distant metastasis in a zebrafish xenograft model. Representative fluorescence (Magenta) images of U-hth-104 cells injected into perivitelline space (red arrow) of zebrafish larvae 48 h post fertilization under 10X objective. White arrow marks distant tumor foci quantified after 24 h post injection. Scale bar denotes 200 μm (**H**) Numbers of tumor foci found in the tails of zebrafish larvae. (**I**) Downregulation of *DICER1* mRNA expression in GABPB1-depleted cells. C: control; GA: GABPA; GB: GABPB1; ns: not significant; no: number.

Our recent study showed that GABPA knockdown leads to diminished *TERT* expression, whereas it facilitates the invasive phenotype in TC cells [27]. To determine if this is also the case for GABPB1, the partner of GABPA, we inhibited GABPB1 expression in U-hth-74 and U-hth-104 cells using RNA interference (RNAi) technique and, at the same time, included GABPA RNAi as an additional control to further verify the specificity of these siRNAs (Figure 1A). By downregulating GABPB1, these TC cells displayed significantly reduced *TERT* expression in both U-hth-74 (1.00 ± 0.01 vs. 0.28 ± 0.23 for control and GABPB1 RNAis, respectively, *p* = 0.006) and U-hth-104 cells (1.01 ± 0.01 vs. 0.17 ± 0.04 for control and GABPB1 RNAis, respectively, *p* < 0.001) (Figure 1B). However, knocking down GABPB1 did not influence the proliferation of U-hth-74 and U-hth-104 (Figure 1C,D) or apoptosis of U-hth-104 cells (Figure S1A). On the other hand, these same sets of cells exhibited a robust increase in invasive capacity, as determined using matrigel assays (Figure 1E,F). We further investigated if the metastatic potential was affected after downregulating GABPB1 in U-hth-104 cells using a zebrafish xenograft model in which engrafted tumor cells need to invade through the perivitelline space and enter the blood circulation of the organism for formation of distant metastasis. We observed that there were indeed more distant metastases with cells bearing siRNA for GABPB1 (Figure 1G,H).

While it was previously demonstrated that GABPA inhibits the invasive phenotype of TC cells by stimulating *DICER1* transcription [27,33], we further analyzed *DICER1* mRNA levels in GABPB1-depleted cells. Indeed, a significant decrease in *DICER1* expression occurred in GABPB1-depleted cells (U-hth-74: 1.01 ± 0.01 vs. 0.57 ± 0.21, *p* = 0.026; U-hth-104: 1.00 ± 0.01 vs. 0.27 ± 0.2, *p* < 0.001) (Figure 1I). Since the invasive phenotype might be related to the induction of EMT, we investigated the expression of vimentin and E-cadherin via flow cytometry. Since the ATC-derived U-hth-104 cells readily expressed very high levels of vimentin (Figure S2A), we used the PTC-derived MDA-T41 cell line with low basal vimentin expression for further studies. Interestingly in MDA-T41, we observed upregulation of vimentin protein expression and downregulation of E-cadherin expression upon knocking down GABPB1 (Figure S2). Similarly, in MDA-T41, knocking down of GABPB1 also resulted in increased invasion in a similar transwell assay setting (Figure S3) but no effect on apoptosis (Figure S1B). Taken together, the observed enhanced invasiveness may be explained by EMT at least partly.

Here, we demonstrated how the depletion of GABPB1 influences the invasiveness and migration of TC cells and that there is a functional similarity between GABPB1 and GABPA in altering the gene expressions of *TERT* and *DICER1*.

### 3.2. The Association between Low GABPB1 Expression and Aggressiveness in TC

**Figure 2:**
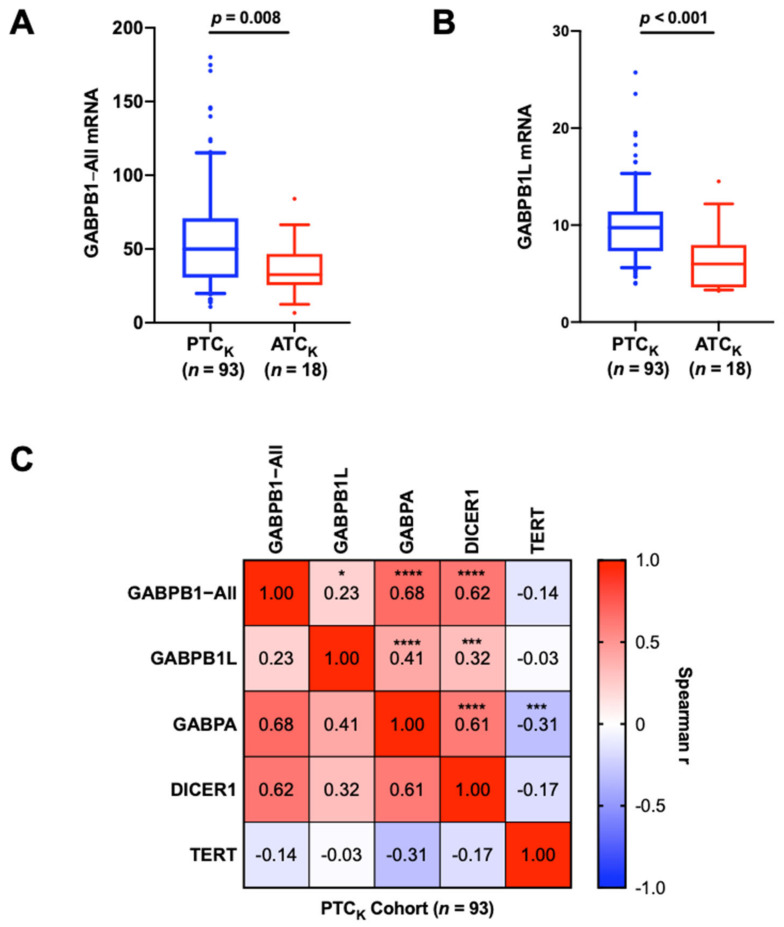
*GABPB1* mRNA expression levels are associated with aggressive TCs, and positively correlated with *GABPA* and *DICER1* expression. Gene expression levels were determined using qPCR. (**A**,**B**) Significantly lower expression of *GABPB1*−*All* and *GABPB1L* in ATC_K_ than in PTC_K_, respectively. (**C**) Correlation matrix for analyses between *GABPB1−All*, *GABPB1L*, *GABPA*, *DICER1,* and *TERT* mRNA levels in PTC_K_. Spearman correlation r values are illustrated in color from blue (−1) to red (1). *GABPB1−All*: total amount of *GABPB1* mRNAs; including all transcript variants of *GABPB1*; *GABPB1L:* long variant of *GABPB1* mRNA; * *p* < 0.05; *** *p* < 0.001; **** *p* < 0.0001.

The findings above suggest that GABPB1 is required for constitutive *TERT* expression, whereas it may itself suppress the invasiveness of TC cells. To investigate further, we compared the gene expression levels of *GABPB1* between primary tumors from PTC_K_ and ATC_K_ using qPCR. The total amount of *GABPB1* mRNAs (*GABPB1−All*; including all transcript variants by detecting the abundance of the shared region among different *GABPB1* variants) was significantly lower in ATC_K_ than in PTC_K_ (PTC_K_ vs. ATC_K_: 58.66 ± 37.64 vs. 36.08 ± 18.62, *p* = 0.008) (Figure 2A). Since the long variant of *GABPB1* mRNA (*GABPB1L*) but not its shorter transcripts was observed to play a functional role in the activation of the mutated *TERT* promoter in glioblastoma [13], we further determined *GABPB1L* expression in the same tumors. Similarly, a downregulation of *GABPB1L* in ATC_K_ tumors was revealed (PTC_K_ vs. ATC_K_: 10.05 ± 4.0 vs. 6.54 ± 3.37, *p* = 0.0001) (Figure 2B).

Given a tight relationship between *GABPB1*, *GABPA*, *TERT,* and *DICER1*, we sought to determine if there was any correlation between their expression levels in PTC tumors. We also analyzed the relationship between the abundances of *GABPB1−All* and *GABPB1L* transcripts. As shown in Figure 2C, the *GABPB1−All* and *GABPB1L* mRNA levels were highly correlated with each other in the PTC_K_ cohort. In addition, *GABPB1−All* and *GABPB1L* were both correlated with *GABPA* and *DICER1* expression levels. Similar positive correlations were found from the analysis of the PTC_TCGA_ cohort (Figure S4A,B, Table S2). *GABPB1−All* and *GABPA* were also positively correlated in the ATC_K_ cohort (Table S3). *GABPB1* mRNA was also positively correlated with *TERT* mRNA expression in PTC_TCGA_ (Figure S4C and Table S2) but not in PTC_K_ (Table 1). Moreover, as the *BRAF*V600E mutation was recently shown to up-regulate GABPB1 expression [24], we further compared *GABPB1* expression between *BRAF*wt and *BRAF*V600E tumors but found no significant difference in the PTC_K_ or PTC_TCGA_ cohorts (Table 1 and Table S2). Our clinical analysis was indicative that the loss of *GABPB1* gene expression could potentially contribute to tumor progression, highlighting the need for further understanding of the regulatory mechanism underlying its gene expression.

### 3.3. The Methylation Landscape of the GABPB1 Locus across TCGA Pan-Cancer

**Figure 3:**
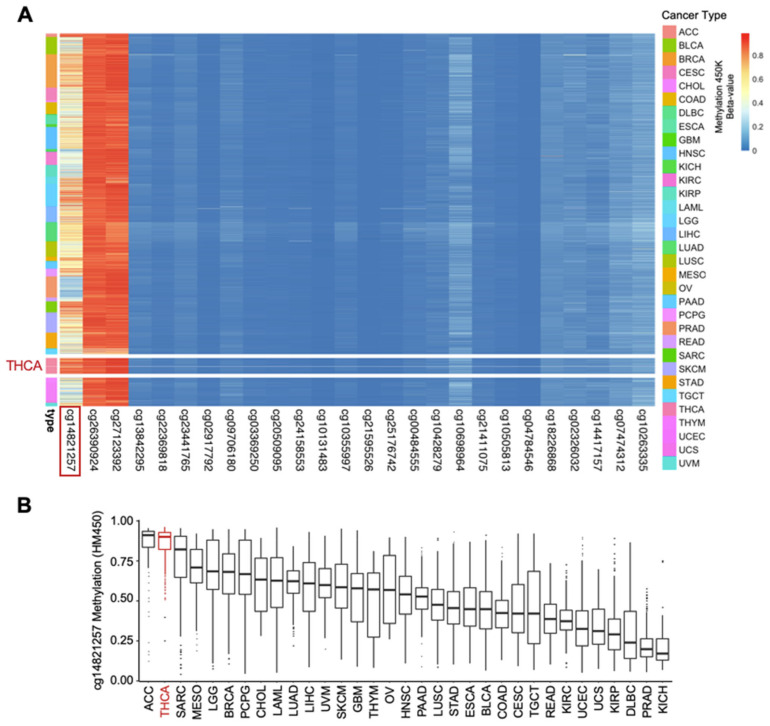
The methylation landscape of the *GABPB1* loci across TCGA Pan-cancer. (**A**) Unclustered heatmap showing the methylation density (β value) of the *GABPB1* gene locus at various CpG sites based on methylation 450K across 33 cancer types. (**B**) Methylation of cg14821257 CpG site. Data are presented in Tukey boxplots with ranked order of cancer types based on median β value.

One of the potential mechanisms underlying reduced *GABPB1* expression in aggressive TCs could be epigenetic regulation. Using a pan-cancer approach, the DNA methylation landscape of the *GABPB1* locus was characterized in TCGA data sets. Across 33 cancer types, three major CpG sites were frequently hypermethylated, but only the cg14821257 site displayed high variability across different cancer types (Figure 3A). Among the 33 cancer types, the top three ranked cancers based on the median of β values of cg14821257 were ACC (adrenocortical carcinoma), THCA (thyroid cancer), and SARC (sarcoma), respectively (Figure 3B). By looking at the average β value across all CpG sites of *GABPB1*, THCA is ranked second following LUAD (lung adenocarcinoma) (Figure S5A). With a rank of 28th out of 33 cancer types, THCA is one of the cancer cohorts with the lowest *GABPB1* mRNA expression (Figure S5B). Based on our pan-cancer analysis, the hypermethylation of cg14821257 of the *GABPB1* gene may have a unique functional role to be further uncovered within thyroid cancer.

### 3.4. Hypermethylation of GABPB1 Promoter Resulted in GABPB1 Downregulation and Was Associated with Shorter Disease-Free Survival

**Figure 4:**
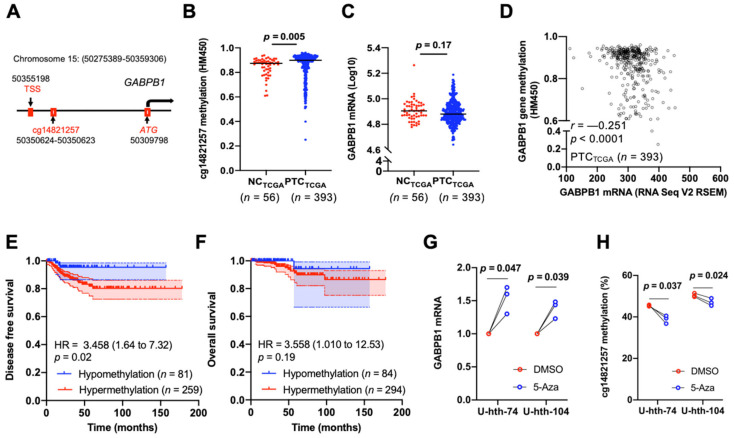
*GABPB1* promoter hypermethylation contributes to the downregulation of *GABPB1* expression and predicts shorter disease-free survival in thyroid cancer. Analyses of *GABPB1* promoter methylation density, gene expression, and survival in PTC_TCGA_ were performed. Cellular experiments using a DNA methylation inhibitor were performed to verify the causal relationship between *GABPB1* promoter methylation and gene expression. (**A**–**D**) Identification of cg14821257 at the *GABPB1* promoter as the predominant CpG affecting *GABPB1* expression. (**B**,**C**) Significantly increased cg14821257 methylation in PTC_TCGA_ tumors as compared to adjacent non-cancerous thyroid tissues (NC_TCGA_), but no significant difference for *GABPB1* expression. (**D**) The inverse correlation between methylation of cg14821257 and *GABPB1* mRNA levels. (**E**,**F**) Hypermethylation of the *GABPB1* promoter was significantly associated with shorter disease-free survival but not with overall survival. (**G**) Upregulation of *GABPB1* expression in U-hth-74 and U-hth-104 cells treated with the DNA methylation inhibitor 5-Aza. (**H**) Reduced cg14821257 methylation in U-hth-74 and U-hth-104 cells treated with the DNA methylation inhibitor 5-Aza. cg14821257 methylation was determined using pyrosequencing.

With the observed hypermethylation of *GABPB1* in PTC from our TCGA pan-cancer analysis, we then investigated the effects of *GABPB1* hypermethylation within PTC. Comparing non-cancerous thyroid tissues and tumors, only cg14821257, a CpG site within the *GABPB1* promoter, was significantly hypermethylated in tumors (Figure S6 and Figure 4A,B). We further compared the *GABPB1* mRNA expression levels between PTC_TCGA_ and NC_TCGA_, but we did not observe a difference between the two groups (Figure 4C). However, a significant inverse correlation between *GABPB1* gene methylation and its mRNA level was observed (r = −0.251, *p* < 0.0001) (Figure 4D). We also determined the correlation between *GABPB1* expression and various CpG sites. cg14821257 was found most significantly correlated with *GABPB1* expression (Table S4). Furthermore, patients with hypermethylated *GABPB1* promoter at cg14821257 showed significantly shorter disease-free survival (Figure 4E and Table S5) but not overall survival (Figure 4F and Table S5) using a β value cut-off at 0.8. We then sought to directly address the relationship between DNA methylation and *GABPB1* expression. U-hth-74 and U-hth-104 cells were treated with the DNA methylation inhibitor 5-Aza, and the methylation density of cg14821257 and *GABPB1* expression were then determined. As shown in Figure 4G,H, 5-Aza treatment led to a significant upregulation of *GABPB1* expression coupled with diminished methylation of cg14821257 in U-hth-74 and U-hth-104 cells as compared with those in control cells incubated with DMSO. Collectively, these data suggested that the increased DNA methylation density exerts its effect on the downregulation of *GABPB1*.

### 3.5. Association of GABPB1 Expression with Clinico-Pathological Variables in TC Tumors

Finally, the relationship between *GABPB1* expression and clinical variables in the PTC_K_, ATC_K,_ and PTC_TCGA_ cohorts was explored. The levels of *GABPB1−All* mRNA were inversely correlated with patient age in PTC_K_ (r = −0.258, *p* = 0.013) (Table 1), and similarly, *GABPB1* mRNA expression was inversely correlated with patient age in PTC_TCGA_ (Table S2). No associations were observed between *GABPB1−All*/*GABPB1* mRNA expression and metastasis and survival in the PTC_K_, ATC_K,_ and PTC_TCGA_ cohort, respectively (Table 1, Tables S2 and S3). None of the above variables was correlated with *GABPB1L* mRNA expression (Table 1 and Table S3).

## 4. Discussion

The *TERT* promoter mutation, a novel mechanism for telomerase activation, occurs frequently in TCs, including PTCs and ATCs [16]. GABPA and GABPB1 have been shown to play a pivotal role in the activation of mutated *TERT* promoters [10,11]. More importantly, the oncogenic *BRAF*V600E up-regulates GABPB1 expression, amplifying the effect on *TERT* transcription [24]. Consistently, the co-occurrence of both *TERT* promoter mutations and *BRAF*V600E is related to PTCs with the poorest clinical outcomes [17]. However, to our surprise, the present findings show that GABPB1 knockdown reduces *TERT* expression while enhancing the invasiveness of TC cells and metastatic potential in a xenograft zebrafish model.

We have recently demonstrated that inhibition of GABPA, the interacting partner of GABPB1, similarly facilitates cellular invasion in TCs [27]. GABPA over-expression inhibits in vivo metastasis in xenograft murine models, while the lower expression of *GABPA* is associated with aggressive disease and poor outcomes in PTC patients [27]. Mechanistically, GABPA stimulates the transcription of its target gene, *DICER1,* through which metastasis is suppressed [27,33]. In the same PTC_K_ and PTC_TCGA_ cohort of tumors, *GABPB1* and *DICER1* expression were also highly correlated, suggesting a mechanistic link between GABPA and GABPB1 in the regulation of invasive phenotypes. We also demonstrated that the invasive phenotype might be caused by the induction of EMT. Upregulation of vimentin protein expression and downregulation of E-cadherin expression were observed upon knocking down GABPB1.

GABPB1, encoded by a single gene at chromosome 15q21, exists in four isoforms, including two long (GABPB1L) and two short (GABPB1S) members [11]. Mancini et al. showed that the expression of GABPB1L rather than GABPB1S was positively correlated to *TERT* mRNA levels in primary glioblastoma tumors and that GABPB1L was responsible for the activation of the mutated *TERT* promoter [13]. It is known that GABPB1L harbors highly homologous C-terminal leucine-zipper domains (LZD), mediating its homodimerization or heterodimerization, while GABPB1S does not [11]. Moreover, to activate target genes, GABPB1 needs to form the complex with its partner GABPA, and the heterotetramerization of two GABPA-GABPB1 complexes via LZD is the superb form for transcriptional activities [11]. These structural differences between GABPB1L and GABPB1S provide a putative explanation for the observations in glioblastomas mentioned above. In the present study, however, we did not see any associations of *TERT* expression with either *GABPB1L* or the *GABPB1−All* expression in PTC_K_ tumors, although knocking down GABPB1 led to diminished *TERT* expression in TC cell lines harboring *TERT* promoter mutation. Intriguingly, we observed an inverse correlation between *GABPA* and *TERT* expression in this same cohort of PTCs, although GABPA is required for activation of the mutated *TERT* promoter. In contrast, the expression of *DICER1*, the target gene of GABPA, is significantly correlated with both *GABPB1L* and *GABPB1−All*. It is thus conceivable that the relationship between GABPA or GABPB1 and TERT is more complicated, and further studies are required to address this issue.

The underlying mechanism in which GABPB1 is involved in suppressing tumor progression remains elusive and seems to be independent of *BRAF*V600E mutation. It was previously shown that the *BRAF*V600E mutation could enhance the expression of GABPB1 [24], but we did not see a significant association of *GABPB1* gene expression and *BRAF*V600E within our clinical analyses of PTC_K_ and PTC_TCGA_, which was further supported by two other recent studies [34,35]. Instead, the hypermethylation of the *GABPB1* promoter was inversely correlated with its expression independently of the presence of *BRAF*V600E, which indicated an epigenetic control of the *GABPB1* regulation. Consistently, inhibiting DNA methylation indeed up-regulated *GABPB1* expression coupled with the reduced *GABPB1* promoter methylation in TC cells. The aberrant DNA methylation is a common mechanism to silence tumor suppressor genes, and here we observed that hypermethylation of *GABPB1* promoter is prognostic for shorter disease-free survival besides an inhibitory effect of GABPB1 on TC cell invasion. As shown previously, the DNA hypermethylation similarly occurs in the *GABPA* promoter, which leads to the diminished *GABPA* expression, thereby attenuating its tumor suppressor function [36].

Given all the oncogenic effects of TERT, GABPB1 and GABPA-mediated *TERT* upregulation is expected to promote cancer progression. However, paradoxically, either GABPB1 or GABPA knockdown contributes to aggressive disease, despite reduced *TERT* expression. Evidently, the loss of GABPB1 or GABPA is a more potent cancer-driving force that compensates for the lack of *TERT* upregulation. However, Liu et al. observed that FOS inhibitor-mediated reduction in GABPB1 and TERT expression induced widespread apoptosis of TC and other cancer cells due to the downregulation of survivin expression, and they further identified TERT as a direct activator for the *survivin* gene transcription [25]. In contrast, we directly knocked down GABPB1 in TC cells but observed no difference in proliferation and apoptosis despite the diminished *TERT* expression. The contrasting results could be caused by the different cell lines, which calls for further investigations. Moreover, the present study highlighted that the tumor-suppressive functions of GABPB1 may be independent of the *TERT* promoter since we found a similar enhanced invasive ability after inhibition of GABPB1 in TC cells regardless of their *TERT* promoter mutation status.

## 5. Conclusions

The present findings demonstrate that GABPB1 activates *TERT* expression on one hand, while it inhibits invasion and progression of TC on the other, which is very similar to its partner GABPA. Likely, GABPB1 has a tumor-suppressive function in the TC pathogenesis to counteract the development of aggressive diseases. Therefore, targeting GABPB1 for anti-telomerase cancer therapy may counteract its tumor-suppressive activity, thereby promoting TC progression, which should be considered in the rational development of telomerase-based strategies against cancer.

## Figures and Tables

**Table 1:** Clinical characteristics and statistical comparison for the 93 PTC_K_ cases in the study.

Parameter (*n* = Informative)	Observations	*GABPB1*−*All* mRNA	*GABPB1L* mRNA
Age at diagnosis (*n* = 93)		r = −0.258, *p* = 0.013	r = −0.171, *p* = 0.101
Median (min–max) yrs	51 (15–97)		
Sex (*n* = 93)		*p* = 0.751	*p* = 0.262
Female/Male	*n* = 67/*n* = 26		
Tumor size (*n* = 88)		r = −0.183, *p* = 0.089	r = −0.109, *p* = 0.313
Median (min–max) cm	2.5 (0.3–12)		
Lymph node metastasis (*n* = 93)		*p* = 0.114	*p* = 0.627
Yes	*n* = 49		
No	*n* = 44		
Distant metastasis (*n* = 93)		*p* = 0.176	*p* = 0.973
Yes	*n* = 12		
No	*n* = 81		
*BRAF* V600E (*n* = 93)		*p* = 0.119	*p* = 0.454
Mutation	*n* = 70		
Wild-type	*n* = 23		
*TERT* promoter mutation (*n* = 93)		*p* = 0.007	*p* = 0.154
Mutation	*n* = 29		
C228T/C250T	*n* = 24/*n* = 5		
Wild-type	*n* = 64		
*TERT* mRNA (*n* = 93)		r = −0.137, *p* = 0.191	r = −0.024, *p* = 0.818
Median (min–max)	0.01 (0.00–12.3)		
*GABPA* mRNA (*n* = 93)		r = 0.676, *p* < 0.001	r = 0.410, *p* < 0.001
Median (min–max)	6.8 (0.2–15.9)		
*DICER1* mRNA (*n* = 93)		r = 0.624, *p* < 0.001	r = 0.321, *p* = 0.002
Median (min–max)	19.8 (1.3–124.6)		
Overall survival (*n* = 93)		* HR = 0.998, *p* = 0.674	* HR = 0.930, *p* = 0.191
Dead	*n* = 32	95% CI = 0.998–1.008	95% CI = 0.835–1.037
Alive	*n* = 61		
Follow-up: median (min–max) yrs	14.8 (0.2–26.5)		
Disease-free survival (*n* = 93)		* HR = 0.994, *p* = 0.314	* HR = 0.952, *p* = 0.391
Relapsed/progression	*n* = 26	95% CI = 0.982–1.006	95% CI = 0.850–1.066
No evidence of disease	*n* = 67		
Follow-up: median (min–max) yrs	13.5 (0.1–26.5)		

Mann–Whitney *U*-test was used for comparison between groups. Spearman’s rank-order correlation r was used for correlation. Univariate Cox regression was used for survival analyses. * HR for *GABPB1−All/GABPB1L* mRNA expression as a continuous variable. Abbreviations: *GABPB1−All* = total amount of *GABPB1* mRNAs, including all transcript variants of *GABPB1*; *GABPB1L =* long variant of *GABPB1* mRNA.*n* = number; yrs = years; HR = hazard ratio; 95% CI = 95% confidence interval.

## Supplementary Materials

The following supplementary data are available online at https://www.mdpi.com/article/10.3390/cancers14061385/s1. Figure S1: Survival of thyroid cancer cells are not affected by knocking down GABPB1. Figure S2: Knocking down GABPB1 expression induces EMT phenotype in MDA-T41 cells. Figure S3: Knocking down GABPB1 expression enhances invasiveness of MDA-T41 cells. Figure S4: GABPB1 expression is positively correlated with GABPA, DICER1, and TERT expression in PTC_TCGA_ cohort (A, B, and C). Figure S5: DNA methylation and mRNA expression of GABPB1 across TCGA Pan-Cancer. Figure S6: Methylation density of CpG sites within GABPB1 gene locus. Figure S7. Original Images for Western blots (Figure 1A). Table S1: Sequences of primers and RNA used in the study. Table S2: Clinical characteristics and statistical comparison for the 393 cases in PTC_TCGA_. Table S3: Clinical characteristics and statistical comparison for the 18 ATC_K_ cases. Table S4: Various CpG sites at the *GABPB1* locus and their correlation with GABPB1 mRNA expression in PTC_TCGA_. Table S5: Correlation between GABPB1 promoter methylation density and clinical characteristics in PTC_TCGA_.
