# Supplementary material for: Downregulation and Hypermethylation of GABPB1 Is Associated with Aggressive Thyroid Cancer Features
Source: Cancers (Basel). 2022 Mar 8;14(6):1385. doi: 10.3390/cancers14061385 (PMC8946831; doi:10.3390/cancers14061385)
Supplement: Supplementary file 1 [file cancers-14-01385-s001.zip › cancers-1494560-supplementary.pdf]

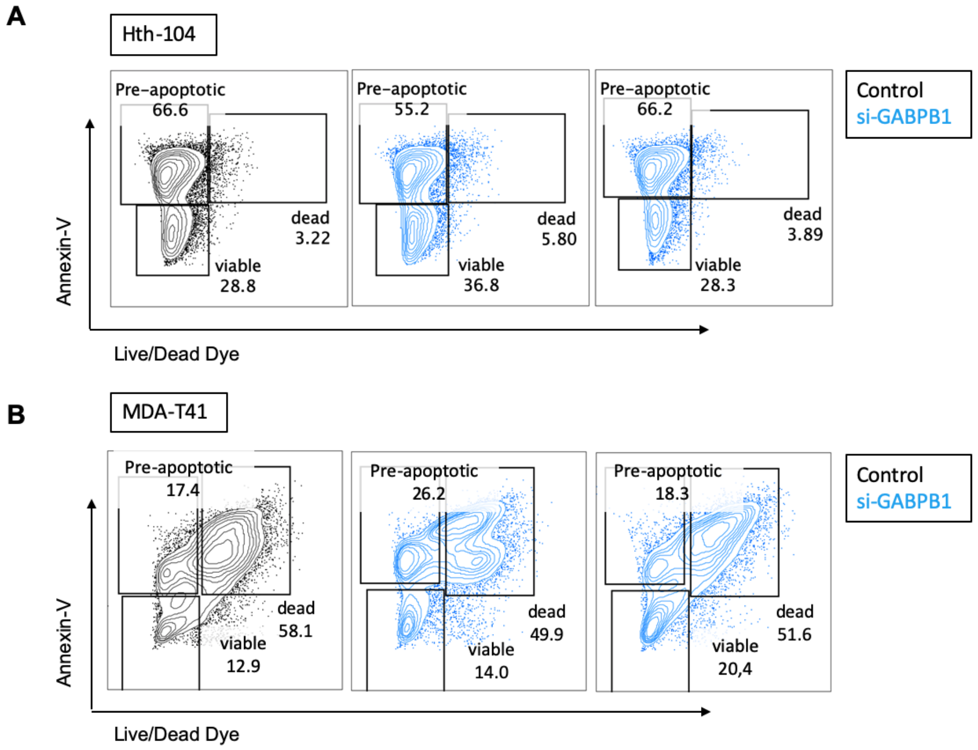

Figure S1. Survival of thyroid cancer cells are not affected after knocking down GABPB1. Flow cytometric plots of (**A**) U-hth-104 cells and (**B**) MDA-T41 cells showed surface expression of Annexin-V and uptake of near-IR live-dead marker dye. Plots in blue are cells with si-GABPB1.

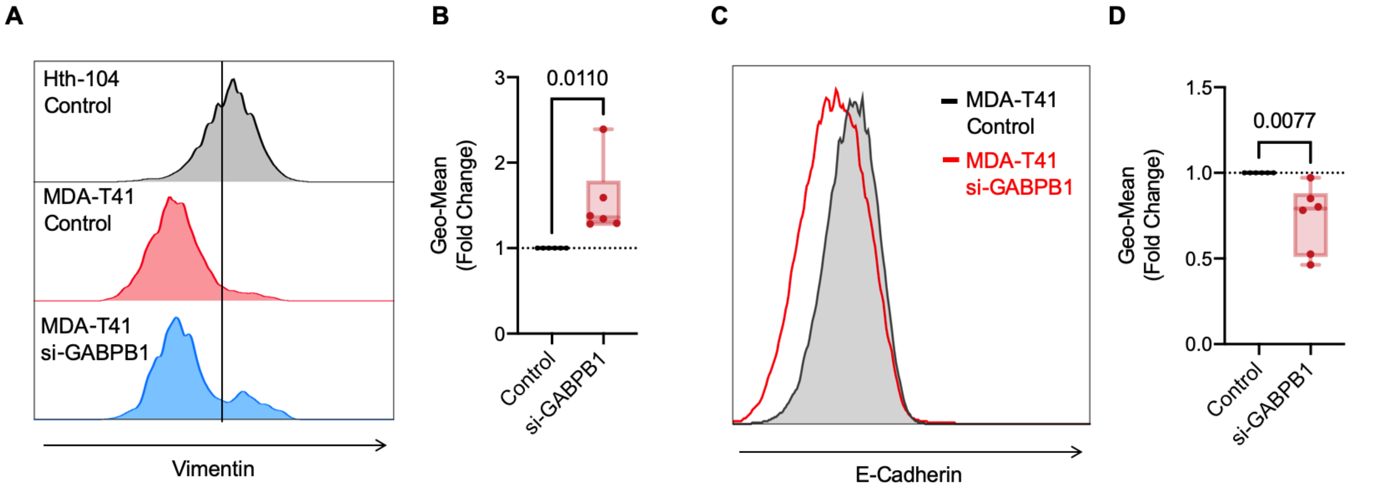

Figure S2. Knocking down GABPB1 expression induces epithelial-to-mesenchymal transition (EMT) phenotype in MDA-T41 cells. (**A**) Representative histogram of vimentin expression changes in MDA-T41 with U-hth-104 cells as a positive control. **(B)** Fold change quantification of geometric mean of fluorescence intensity for vimentin FACS staining. (**C**) Representative histogram of E-cadherin expression changes in MDA-T41. (**D**) Fold change quantification of geometric mean of fluorescence intensity for E-cadherin FACS staining. (**B**) and (**D**) Student *t*-test was used to test for significance with sample size of $n = 6$.

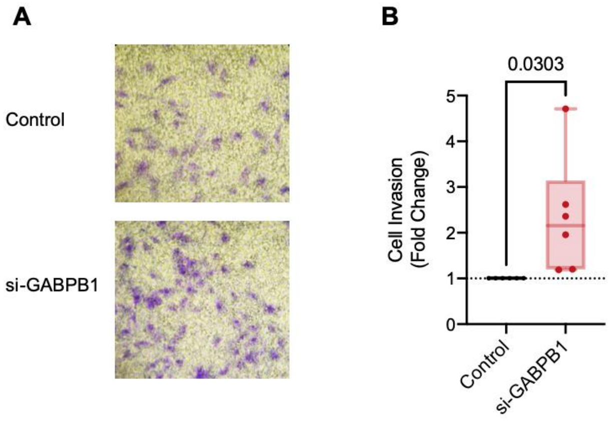

Figure S3. Knocking down GABPB1 expression enhances invasiveness of MDA-T41 cells**.** (**A**) Representative images of invaded MDA-T41 cells in a transwell assay. (**B**) Fold change quantification of invaded cells comparing MDA-T41 bearing siRNA for GABPB1 versus control. Student *t*-test was used to test for significance with sample size $n = 6$.

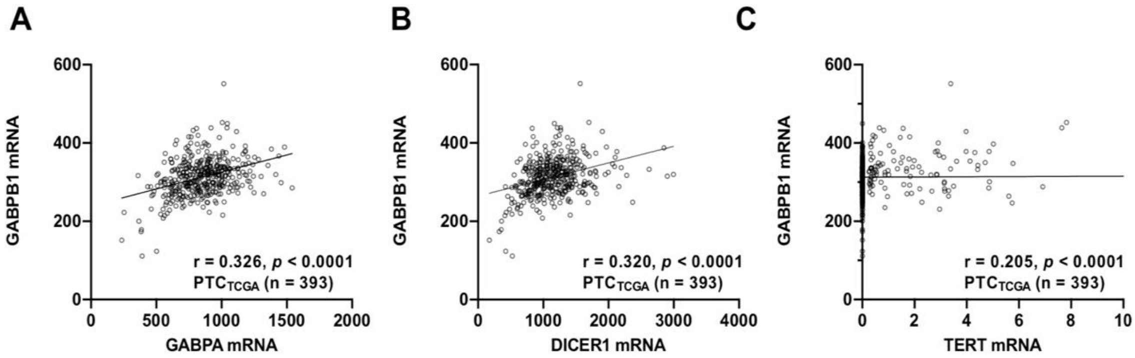

Figure S4. *GABPB1* expression is positively correlated with *GABPA*, *DICER1* and *TERT* expression in PTC_{TCGA} cohort (**A**, **B** and **C**). mRNA abundances were expressed as RSEM (RNA-Seq by Expectation Maximization).

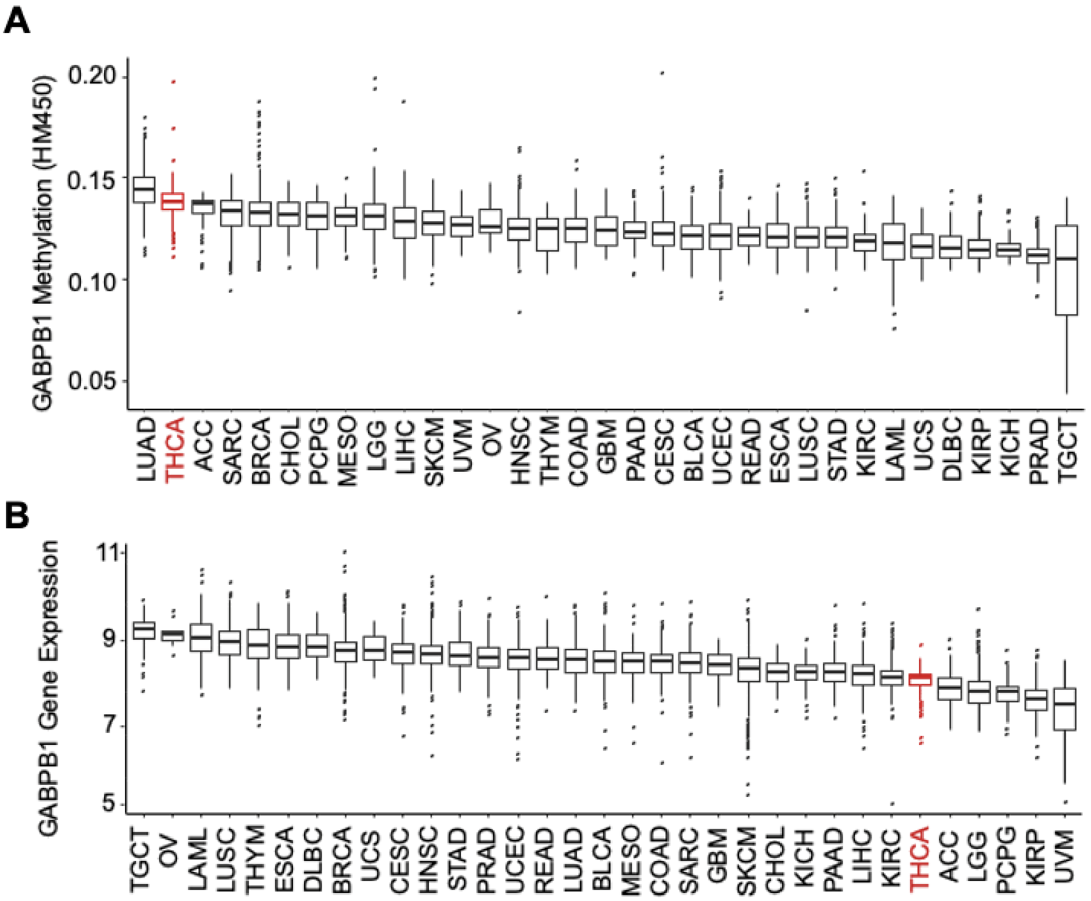

Figure S5. DNA Methylation and mRNA expression of *GABPB1* across TCGA Pan-Cancer. (**A**) Average methylation density of all CpG sites within the *GABPB1* gene locus across 33 cancer types in TCGA Pan-cancer. (**B**) Normalized gene expression of GABPB1 across 33 cancer types in TCGA Pan-cancer. All data presented in Tukey boxplots with order of cancer types ranked based on median value.

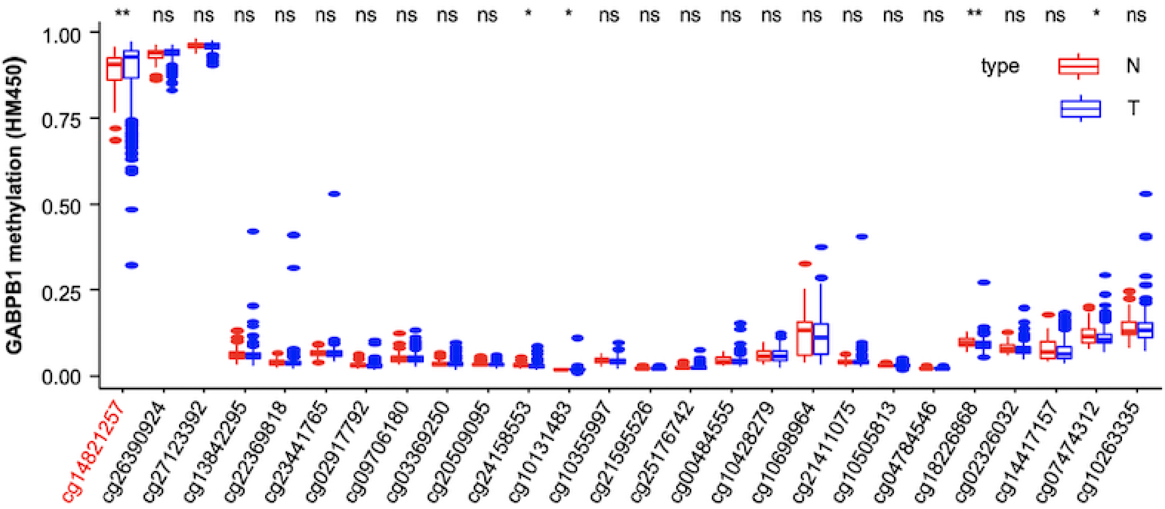

Figure S6. Methylation Density of CpG sites within *GABPB1* gene locus**.** N: NC_{TCGA}, T: PTC_{TCGA}, ns: not significant, * $p < 0.05$, ** $p < 0.01$.

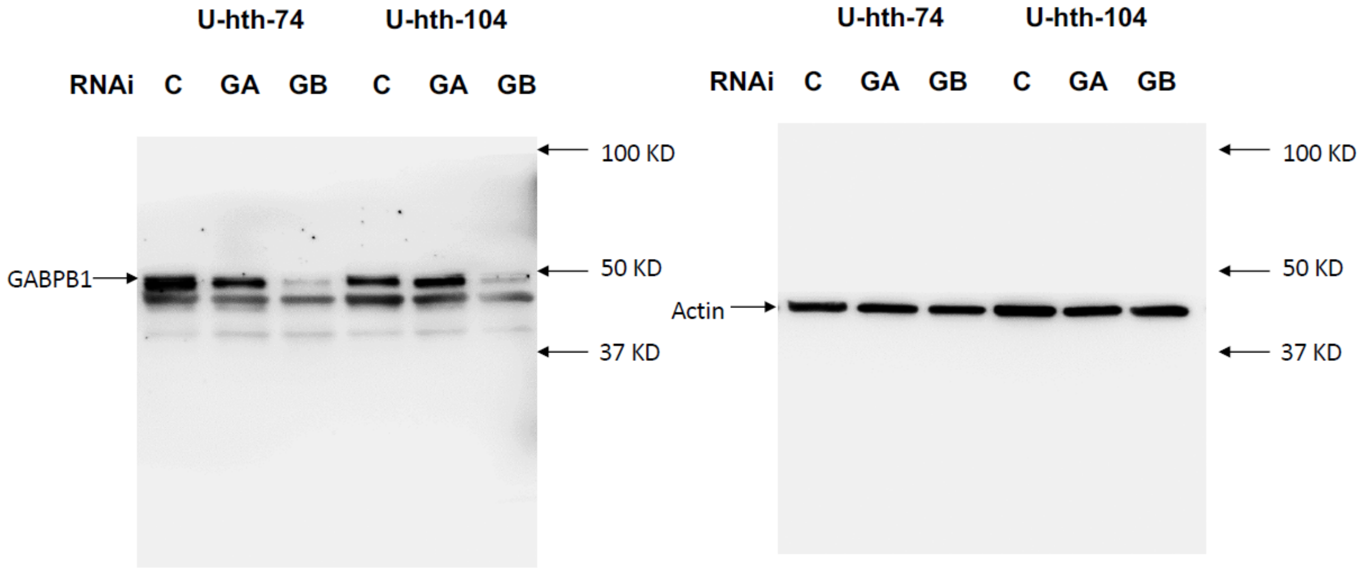

Figure S7. Original Images for Western blots (Figure 1A).

Table S1. Sequences of primers and RNAis used in the study.

Target	Direction	Sequence
Sanger sequencing		
TERT promoter mutation	Forward	5′-CACCCGTCCTGCCCCTTCACCTT-3′
	Reverse	5′-GGCTTCCCACGTGCGCAGCAGGA-3′
BRAF V600E	Forward	5′-GCTTGCTCTGATAGGAAAATGAG-3′
	Reverse	5′-GTAACTCAGCAGCATCTCAGG-3′
Pyrosequencing		
GABPB1 PCR	Forward	5′- TGGGGATTTTAGAGTAGAGGAA -3′
	Reverse	5′-Biotin- ACTCAATTAACTTTTTATTCTTCCACCTAT -3′
GABPB1 Sequencing		5′- AATTTTGAGGTTTTATTAAATATGT -3′
Quantitative Real-time PCR		
TERT	Forward	5′-CGGAAGAGTGTCTGGAGCAA-3′
	Reverse	5′-GGATGAAGCGGAGTCTGGA-3′
GABPA	Forward	5′- AAGAACGCCTTGGGATACCCT-3′
	Reverse	5′- GTGAGGTCTATATCGGTCATGCT-3′
DICER1	Forward	5′-CCTAGACCACCCCTATCGAGA-3′
	Reverse	5′-CAGGTCAGTTGCAGTTTCAGCA-3′
B2M (β2-M)	Forward	5′-GAATTGCTATGTGTCTGGGT-3′
	Reverse	5′-CATCTTCAAACCTCCATGATG-3′
ACTB (β-actin)	Forward	5′-GCGGGAAATCGTGCGTGACAT -3′
	Reverse	5′-TGGCGTACAGGTCTTTGCGGATG-3′
GABPB1-All	Forward	5′-GACCTGGAGGGGTGGTGA-3′
	Reverse	5′-GGCCACTACTGGAGTTTCTGA-3′
GABPB1L	Forward	5′-ATTGAAAACCGGGTGGAA-3′
	Reverse	5′-CTGTAGGCCTCTGCTTCCT-3′
siRNA		
GABPA		5′-GGAGCUGAUAGAAAUUGAGAUUGAU-3′
GABPB1		5′-GAAACUCUUCUACAUCAGUAUUAGC-3′

Table S2. Clinical characteristics and statistical comparison for the 393 cases in PTC_{TCGA}.

**Parameter (*n* = Informative)**	**Observations**	***GABPB1* mRNA**
Age at diagnosis (*n* = 393)		r = -0.201, *p* < 0.0001
median (min-max) yrs	46 (15–89)	
Gender (*n* = 393)		*p* = 0.799
Female / Male	*n* = 285 / *n* = 108	
Lymph node metastasis (*n* = 367)		*p* = 0.072
Yes	*n* = 206	
No	*n* = 161	
Distant metastasis (*n* = 249)		*p* = 0.894
Yes	*n* = 4	
No	*n* = 245	
BRAF V600E (*n* = 310)		*p* = 0.692
Mutation	*n* = 219	
Wild-type	*n* = 91	
TERT promoter mutation (*n* = 312)		*p* = 0.951
Mutation	*n* = 31	
Wild-type	*n* = 281	
TERT mRNA (*n* = 393)		r = 0.205, *p* < 0.0001
median (min-max)	0.0 (0.0–103.4)	
GABPA mRNA (*n* = 393)		r = 0.326, *p*< 0.0001
median (min-max)	846 (235–1541)	
DICER1 mRNA (*n* = 393)		r = 0.320, *p* < 0.0001
median (min-max)	1134 (173–2996)	
Overall survival (*n* = 393)		**HR = 0.993, *p* = 0.122
Dead	*n* = 15	95% CI = 0.985–1.002
Alive	*n* = 378	
Follow-up: median (min-max) months	31.3 (0.0–178.1)	
Disease-free survival (*n* = 380)		**HR = 1.003, *p* = 0.444
Relapsed/progression	*n* = 40	95% CI = 0.996–1.009
No evidence of disease	*n* = 340	
Follow-up: median (min-max) months	29.9 (0.0–178.1)	

Mann-Whitney *U*-test was used for comparison between groups; Spearman's Rank-Order Correlation r was used for correlation; Univariate Cox regression was used for survival analyses; ** HR for *GABPB1* mRNA expression as continuous variable; Abbreviations: *n* = number ; yrs = years; HR = Hazard ratio; 95% CI = 95% confidence interval.

Table S3. Clinical characteristics and statistical comparison for the 18 ATCK cases

**Parameter (n=informative)**	**Observations**	***GABPB1-All* mRNA**	***GABPB1L* mRNA**
Age at diagnosis (*n* = 18)		r = 0.044, *p* = 0.861	r = -0.114, *p* = 0.653
median (min-max) yrs	77.5 (54 - 91)		
Gender (*n* = 18)		*p* = 0.722	*p* = 0.790
Female / Male	*n* = 10 / *n* = 8		
Tumor size (*n* = 18)		r = -0.196, *p* = 0.435	r = -0.279, *p* = 0.262
median (min-max) cm	6.8 (2.0 -10.0)		
TERT promoter mutation (*n* = 18)		*p* = 0.594	*p* = 0.477
Mutation	*n* = 10		
C228T / C250T	*n* = 7 / *n*=3		
Wild-type	*n* = 8		
TERT mRNA (*n* = 18)		r = 0.005, *p* = 0.984	r = -0.004, *p* = 0.987
median (min-max)	0.3 (0.0 - 2.7)		
GABPA mRNA (*n* = 18)		r = 0.891, *p* < 0.001	r = 0.439, *p* = 0.069
median (min-max)	2.1 (1.3 - 6.5)		
Survival (*n* = 17)			
Dead	*n* = 16	*p* = 0.153	*p* = 0.838
Alive	*n* = 1		
Follow-up: median (min-max) months	3 (0 - 190)		

Mann-Whitney U-test was used for comparison between groups. Spearman's Rank-Order Correlation r was used for correlation. Abbreviations: n = number.

Table S4. Various CpG sites at the GABPB1 locus and their correlation with GABPB1 mRNA expression in PTC_{TCGA}.

Cpg site	Chromosome	Start	End	Spearman r	*p* value
cg14821257	chr15	50350623	50350624	-0.2509	<0.0001****
cg26390924	chr15	50321505	50321506	0.01273	0.8014
cg27123392	chr15	50278276	50278277	-0.0655	0.1951
cg13842295	chr15	50355659	50355660	0.02136	0.673
cg22369818	chr15	50355625	50355626	0.02722	0.5906
cg23441765	chr15	50355544	50355545	0.05818	0.2499
cg02917792	chr15	50355523	50355524	0.01946	0.7005
cg09706180	chr15	50355512	50355513	-0.001019	0.9839
cg03369250	chr15	50355427	50355428	0.002721	0.9571
cg20509095	chr15	50355421	50355422	-0.02762	0.5852
cg24158553	chr15	50355411	50355412	0.01263	0.8029
cg10131483	chr15	50355386	50355387	-0.08996	0.0749
cg10355997	chr15	50355357	50355358	0.05541	0.2732
cg21595526	chr15	50355070	50355071	-0.03093	0.5409
cg25176742	chr15	50355056	50355057	-0.02493	0.6222
cg00484555	chr15	50355040	50355041	0.1203	0.0171*
cg10428279	chr15	50354997	50354998	0.135	0.0074**
cg10698964	chr15	50354976	50354977	0.1572	0.0018**
cg21411075	chr15	50354899	50354900	-0.01372	0.7866
cg10505813	chr15	50354854	50354855	-0.05042	0.3188
cg04784546	chr15	50354420	50354421	-0.08849	0.0798
cg18226868	chr15	50354241	50354242	0.02273	0.6533
cg02326032	chr15	50354153	50354154	-0.05451	0.281
cg14417157	chr15	50353990	50353991	0.1544	0.0021**
cg07474312	chr15	50353962	50353963	0.09008	0.0745
cg10263335	chr15	50353902	50353903	0.1057	0.0365*

Spearman's Rank-Order Correlation r was used for correlation. * $p < 0.05$, ** $p < 0.01$, **** $p < 0.0001$. Abbreviations: chr15 = chromosome 15.

Table S5. Correlation between *GABPB1* promoter methylation density and clinical characteristics in PTC_{TCGA}.

Parameter (*n* = Informative)	*GABPB1* Promoter Methylation (cg14821257) Hypomethylation (β < 0.8)	Hypermethylation (β ≥ 0.8)	*p* Value
Age at diagnosis (*n* = 393)			0.923
< 55 (*n* = 218)	*n* = 70	*n* = 148	
≥ 55 (*n* = 175)	*n* = 57	*n* = 118	
Gender (*n* = 393)			0.454
Female (*n* = 285)	*n* = 89	*n* = 196	
Male (*n* = 108)	*n* = 38	*n* = 70	
Disease stage (*n* = 392)			0.602
Stage I + II (*n* = 251)	*n* = 79	*n* = 172	
Stage III + IV (*n* = 141)	*n* = 48	*n* = 93	
Lymph node metastasis (*n* = 367)			0.298
Yes (*n* = 206)	*n* = 72	*n* = 134	
No (*n* = 161)	*n* = 48	*n* = 113	
Distant metastasis (*n* = 249)			0.154
Yes (*n* = 4)	*n* = 0	*n* = 4	
No (*n* = 245)	*n* = 83	*n* = 162	
BRAF V600E (*n* = 310)			0.351
Mutation (*n* = 219)	*n* = 77	*n* = 142	
Wild-type (*n* = 91)	*n* = 27	*n* = 64	
TERT promoter mutation (*n* =312)			0.592
Mutation (*n* = 31)	*n* = 9	*n* = 22	
Wild-type (*n* = 281)	*n* = 95	*n* = 186	
Overall survival (*n* = 393)			0.208
Dead (*n* = 15)	*n* = 1	*n* = 14	
Alive (*n* = 378)	*n* = 84	*n* = 294	
Disease-free survival (*n* = 380)			0.023
Relapsed/progression (*n* = 40)	*n* = 3	*n* = 37	
No evidence of disease (*n* = 340)	*n* = 81	*n* = 259	

Chi^2-test or Fisher's exact Test was used for comparison between groups. DNA methylation was expressed as β values (the ratio of signal intensity between methylated and unmethylated CpGs). Abbreviations: n = number.
